# Engineering Student’s Ethical Awareness and Behavior: A New Motivational Model

**DOI:** 10.1007/s11948-016-9814-x

**Published:** 2016-10-17

**Authors:** Diana Bairaktarova, Anna Woodcock

**Affiliations:** 1 Department of Engineering Education, Virginia Polytechnic Institute and State University, Engineering Education (0218), Goodwin Hall, Room 367, 635 Prices Fork Road, Blacksburg, VA 24061 USA; 2 Department of Psychology, California State University San Marcos, San Marcos, CA USA

**Keywords:** Ethical reasoning, Ethical behavior, Engineering ethics education, Motivational model

## Abstract

Professional communities are experiencing scandals involving unethical and illegal practices daily. Yet it should not take a national major structure failure to highlight the importance of ethical awareness and behavior, or the need for the development and practice of ethical behavior in engineering students. Development of ethical behavior skills in future engineers is a key competency for engineering schools as ethical behavior is a part of the professional identity and practice of engineers. While engineering educators have somewhat established instructional methods to teach engineering ethics, they still rely heavily on teaching ethical awareness, and pay little attention to how well ethical awareness predicts ethical behavior. However the ability to exercise ethical judgement does not mean that students are ethically educated or likely to behave in an ethical manner. This paper argues measuring ethical judgment is insufficient for evaluating the teaching of engineering ethics, because ethical awareness has not been demonstrated to translate into ethical behavior. The focus of this paper is to propose a model that correlates with both, ethical awareness and ethical behavior. This model integrates the theory of planned behavior, person and thing orientation, and spheres of control. Applying this model will allow educators to build confidence and trust in their students’ ability to build a professional identity and be prepared for the engineering profession and practice.

## Introduction

The engineering community has experienced numerous scandals involving unethical and illegal engineering practices; many of them committed in large and well-known engineering companies and government agencies. It should not take a national major structure failure to remind us of the importance of ethical awareness and behavior, along with supporting the development and practice of ethical behavior in engineering students. Development of ethical judgment skills in future engineers is a key competency for engineering schools as engineering ethics is part of the engineering thinking, identity and professional practice of engineers (Harris et al. 1996). Future engineering employees are not only expected to have technical knowledge, skills, and abilities, but also a foundation in professional and ethical practices (The Engineer of 2020). Given that engineering undergraduates are expected to enable and sustain industries, and help societies prosper economically (Sheppard et al. 2008), engineering schools have an obligation to provide professional ethics, not merely a technical education for future engineers. The steady growth of technology and innovations makes the oversight of technology far more complex, political, and disruptive than in the past (Falconi 2015).

There is no question of the importance in the education of engineering students developing ethical decision-making abilities of our future engineer leaders and innovators. However, the question engineering educators struggle with is how to best accomplish this goal. The pedagogical framework of engineering ethics education has evolved primarily toward the utilization of case studies, codes of ethics and introduction to moral theory. As educators, we have established instructional methods to teach engineering ethics, however, we still rely heavily on teaching moral judgment and ethical reasoning and pay little attention to how well ethical awareness predicts ethical behavior. The literature reveals that engineering ethics courses have stressed skills acquisition rather than behavior change (Shuman et al. 2004; Burgess et al. 2012). We argue that the ability to exercise moral judgement does not necessarily mean that students are ethically educated or likely to behave in an ethical manner. We claim that measuring ethical reasoning is insufficient for teaching engineering ethics because ethical reasoning has not been demonstrated to translate reliably into ethical behavior.

The focus of this paper is to propose a model that accounts for both ethical awareness (including reasoning and judgment) and ethical behavior. Applying this model will allow educators to build confidence and trust in our students so that they will build their professional identity and are prepared for the engineering profession and practice.

We begin the paper by reviewing the current state of ethics education in the US undergraduate engineering curriculum and discussing the commonly used assessment tools such as Kohlberg’s stages of moral reasoning and Rest’s Defining Issues Test (DIT). We then argue for the need to go beyond measuring ethical reasoning. We propose a model that builds on Rest’s psychological processes of moral sensitivity, moral judgement, moral motivation, and moral character and implementation. This model takes into account students’ attitudes toward ethical behavior, the social norms supporting them to behave ethically, their perceived control over behaving ethically, and their professional obligations. This new model aligns with Fink’s course design approach, which provides a framework to teach and measure the variables in the proposed model: positive attitudes toward ethical behavior, a feeling of support to act ethically, a sense of professional obligation, and awareness of the different spheres of behavioral control and how they relate to supporting ethical behavior. These variables can be easily assessed at the beginning of a course and evaluated throughout. The model also allows for the selection of specific instructional resources and learning activities that tie directly to each student outcome. We hope our model will provide a concise way of looking at and teaching ethics and assessing the components that may predict future ethical behavior.

## Teaching Ethics in Undergraduate Engineering Curricula

With ethics, there is frequently no absolute right answer. It is not straightforward to teach professional ethics or to motivate students to take professional ethics seriously (Bairaktarova and Woodcock 2014). In many engineering curricula ethics is not a required course. However, engineering graduates are still expected to demonstrate that they are ethically and professionally grounded. ABET’s Engineering Criteria 2007 requires engineering programs to demonstrate that their graduates have an understanding of professional and ethical responsibility, but teaching engineering ethics is still not a high priority in engineering education. Evidence from empirical research shows engineering ethics education is not effective. For example, earlier research shows no evidence of a gain in the ethical decision making between freshmen and senior year students (Shuman et al. 2004), which at that time indicated that the undergraduate curriculum is inadequate in preparing students to face ethical issues in their workplace (Shuman et al. 2004; McGinn 2003). The importance of this notion was reaffirmed with a 2006 report by researchers at Penn State University detailing the outcomes of ABET’s EC2000 program pushing for more inclusion of ethical awareness within engineering programs (Lattuca et al. 2006). The study compared the then-current student performance in key accreditation areas and found that awareness of societal and global issues can affect engineering decisions, but issues relating to ethics were among the areas needing improvement.

In a comprehensive review with a diverse sample across the United States engineering schools, a couple years later after the 2006 report, Colby and Sullivan (2008) provided a thorough status of how undergraduate engineering education supports students’ ethical development. Their analysis of the strengths and weaknesses of existing efforts indicated that engineering programs lack accurate and reliable ways of teaching professional ethics and measuring its outcome (Colby and Sullivan 2008). Dedicated engineering ethics courses are not mandatory and a review of the undergraduate engineering curricula points to inconsistent instructional forms (Colby and Sullivan 2008). The most common forms of instructional, pedagogical, and assessment approaches utilized within the undergraduate engineering curricula are summarized in Table 1. The summary is based on Herkert’s (2002) and Colby and Sullivan’s (2008) reviews of the existing engineering ethics education in undergraduate curricula.

**Table 1:** Instructional methods, pedagogical approaches, and assessment

Curricula approach	Content covered	Pedagogy	Assessment
Stand-alone ethics course	First canon of engineering ethics: Public safety, welfare, and protection of the environment	Lecture/discussion: Introduction to code of ethics; introduction to moral theories	Rubric to measure ethical reasoning (Shuman et al.)
Across the curricula ethics (brief discussion)	Honesty: Loyalty to employer and clients/customers	Case studies	PACES (college cheating test) (Harding et al.)
Modules on engineering ethics: capstone; design courses; introduction to engineering course	Fairness: Conflict of interest, intellectual property, unfair competition, discrimination	Service learning (project-based learning)	No grading for the ethics component of the class

The stand-alone ethics course through the semester, with multiple-credit hours, is a model that allows the class content to focus on discipline-specific issues (Herkert 2002). The stand-alone ethics courses are generally offered by philosophy departments with a focus on introduction to moral theories. An across the curriculum course exposes students mainly to discussions about professional responsibility and ethics. These discussions are offered in multiple courses, throughout the undergraduate curriculum, where the course subject allows for such discussions. This method requires a commitment among all engineering faculty to capitalize on ethics discussions within traditional non-ethics focused courses (Weil 2003). Unfortunately, in many courses of this type, the ethics component of the class assignments is not graded, which may undermine their importance and the degree to which students take ethics seriously. Students may also not take ethics courses seriously as it is not one of the primaries in the engineering curriculum (Colby and Sullivan 2008).

The use of case-based instruction methods appears to be the most common pedagogical method within the engineering ethics education. The case studies we discuss in engineering ethics are complex, dynamic, and not always transparent, and this is true in the world of ethics as well as engineering. At the same time, these cases are events that happened to someone else. We ask students to make abstract decisions that in most cases are based on an established code of ethics and intuition of what is right or wrong rather than on personal experience.

Given the piecemeal approach to engineering ethics education and a lack of agreement on the content or approach, it is perhaps not surprising that students are ill-prepared for the ethics portion of the Fundamental Engineering (FE) exam. Regardless of instructional methods or of how ethics are included in the undergraduate curricula, a part of the FE exam is focused on engineering ethics. The questions included in the ethics part of the exam address the following categories of the Code of Ethics: conflict of interest, welfare of society, whistle blowing, and property issues such as patents and innovations. Most current data from the Survey of Engineering Ethical Development (SEED) project wherein results were published from a longitudinal and a broad national survey of student knowledge of ethics as measured by FE type questions reveals that students make no gains in their knowledge of ethics (Harding et al. 2013). Harding and colleagues suggest that the lack of knowledge gains might happen because of students are not receiving instruction on the Code of Ethics or perhaps students do not retain the knowledge.

We offer another plausible explanation for students’ poor performance on the FE exam—it is possible that as educators, we fail to utilize the appropriate methods of ethics instruction and assessment. The instructor of the particular engineering course subject usually makes the decisions about the pedagogical methods. However, the decision for including ethics training in the curricula is generally established at the institutional level (Barry 2009). The predominant methods of curriculum incorporation include: required courses within the discipline, elective courses outside the discipline, across-the-curriculum, and the linking of ethics with society (Herkert 2002; Harding et al. 2009). In Harding and colleagues in the SEED project report several pedagogies were investigated for their effectiveness in educating students about ethics, including the discussed above curriculum and co-curriculum pedagogies. Recommendations were made for the adoption of “alternative pedagogies”, such as a service learning, that “offer an opportunity to integrate ethics directly into the engineering content” (Harding et al. 2009, p. 6). Engineering faculty have also been creative in finding ways of including ethics directly in their subject areas—from providing immersive engagement for community experience through seeking opportunities for learning communities to work on local community projects, to examining and practicing subtle, deliberative arguments in favor of potentially problematic technologies in the context of ethics and writing courses (Zoltowski et al. 2014; Bairaktarova et al. 2015; Moore 2016).

We should consider another challenge—factors such as students’ gender, age, work experience, personality, nationality and cultural background may play a role in ethical decisions (Eagly 1987). Research indicates that women seem to be more sensitive to ethical scenarios (Gilligan 1982; Greenberg 2002) and that work experience plays a larger role than education, as working people are exposed to more ethically challenging situations (Hofstede 1980, 1991). These factors add complexity to teaching ethics and engaging a diverse student body. Students from a variety of educational or cultural backgrounds may have different knowledge, perspectives, practices and norms regarding moral codes of behavior. It is important that the instructor is committed to present class information that is meaningful and useful to all of the students by understanding and accounting for the multiplicity of attitudes and values they hold. When designing the learning objectives for the course, instructors need to consider what would be the enduring understanding from such a course. Students’ development of awareness and confidence in using their ethical judgment later in life should take precedence over memorizing abstract knowledge of ethics to pass the FE or any ethics related exam.

## Moral Reasoning

Measurement and instruction of engineering ethics is heavily influenced by the notion of moral reasoning—the process of making judgments about what is right and wrong. Most contemporary tools to assess moral reasoning use Kohlberg’s framework of six stages of moral reasoning: avoid punishment, self-interest, “good boy” attitude, law and order morality, social contract, principle. The first two stages fall in the first level of what Kohlberg called Preconventional morality (values in external events). The third and fourth stages are categorized in the Second Level of Conventional morality (performing right role). Lastly, stages five and six belong to the Third Level of moral development, called Preconventional morality (shared standards, rights, and duties). Students respond to scenarios depicting ethical dilemmas and their responses are used to categorize them in accordance with Kohlberg’s framework.

A well-known scenario from Kohlberg’s interviews is “The Heinz dilemma” where a man breaks into a pharmacy to steal a drug to save his wife’s life (Kohlberg 1981). This famous example shows how Kohlberg’s framework presents abstract moral development (the value of obeying the law), but not dilemmas requiring moral awareness nor moral behavior. Kohlberg’s conception of moral development is based on thinking and logic, not on feelings for others. Kohlberg also believed that morals were based on age and “wisdom,” rather than real life experience and empathic identification with others. Kohlberg’s assessment focuses on the justification for someone’s reasoning, rather than the evaluation of correct or incorrect responses. Participants are scored based on the relation between their responses to the dilemmas and Kohlberg’s six predefined stages. According to this framework, an individual has reached the highest level of moral reasoning when (1) their judgments are based on abstract principles not necessarily defined by society’s laws, and (2) when they can generalize ethical principles beyond their own interests.

Kohlberg’s moral assessment has been shown to be a valid and reliable assessment of moral development. However, while Kohlberg’s theory of moral development can be used as a common ground that can serve as guidance for individuals, the theory abstractly focuses on the moral development of an individual. His framework does not account for the influence of moral obligation, attitudes towards moral issues of behaviors, or situational factors such as social norms and perceived control over ethical behavior. Furthermore, one may operate on several levels of Kohlberg’s six stages of moral development at the same time. What is even more critical when considering ethics education is the incongruity between moral reasoning and moral behavior; one can think on one level of Kolhberg’s moral reasoning and behave on another.

Based on Kohlberg’s model, Rest (1986) developed the Defining Issues Test (DIT) to assess moral reasoning with five hypothetical moral dilemmas. Students rate and rank the moral dilemmas by importance of the statements. The DIT measures which schemas or preconceived social scripts students bring to the task of moral reasoning. As the student encounters an item that makes sense and also taps into the student’s predominant schema, that item is rated and ranked as highly important. Alternatively, when the student encounters an item that either doesn’t make sense or seems simplistic and unconvincing, the item receives a low rating and is passed over for the next item. Instead of classifying responses into Kohlberg’s six stages, the DIT scores represent the degree to which a student uses the Personal Interest (Stages 2 and 3), Maintaining Norms (Stage 4), or Postconventional (Stages 5 and 6) schemas to guide their responses to the moral dilemmas. The schemas share some similarities with Kohlberg’s framework, but have a number of distinctions. While Kohlberg’s framework measures moral development based purely on someone’s ability to exercise moral judgment, Rest’s motivational model is built considering that the production of moral behavior is intricate and demands at least four different psychological processes: moral sensitivity (interpreting the situation and recognizing a moral issue), moral judgment (judging which ethical actions are morally right or wrong), moral motivation (prioritizing moral values relative to other values and establishing moral intent), and moral character and implementation (having courage, persisting, overcoming distractions, implementing skills) (Rest 1986). Rest argued that each process is conceptually distinct and that success in one does not imply success in any subsequent stage. For example, an individual with a well-developed sense of moral reasoning and sensitivity may not necessarily have strong intentions to act in an ethical or moral manner.

The goal of engineering ethics education is to teach and assess the full spectrum of Rest’s model to encourage and empower ethical behavior, however, the instruction of engineering ethics has relied heavily on just measuring students’ general ethical reasoning. Our search of the literature reveals scant research linking ethical reasoning with ethical behavior. We did uncover a study that reported moral reasoning as assessed by the DIT was associated with cheating behavior whereby individuals low on moral judgment cheated more and sooner than those with higher moral judgment. However, the study also revealed that even those with high moral judgment cheated given sufficient temptation (Malinowski and Smith 1985). We argue that in the spirit of Rest’s model, the measurement and instruction of ethical awareness, intentions, and behavior are what is needed in engineering ethics education, rather than just instruction and assessment of ethical reasoning.

A decade ago, researchers started to consider other psychological variables in the measurement of engineering ethics. In 2004, Magun-Jackson proposed a psychological model based on Kolhberg’s stages of moral development to integrate ethics education in the engineering curriculum (Magun-Jackson 2004). Three conference papers, published in 2005, 2006 and 2007, discussed research efforts to develop an instrument to assess perceptions and attitudes toward cheating among engineering students (PECES-2 Survey on academic integrity) (Finelli et al. 2005, 2007; Harding et al. 2006). In the same time period, Harding and colleagues published their work applying the theory of planned behavior as a model for predicting academic dishonesty using a sample of engineering students (Harding, et al. 2007). This model has also been used to predict cheating (Mayhew et al. 2009). Applying the PACES-2 survey (Harding et al. 2007) and the DIT-2 (Rest et al. 1999) instruments, in their latest work, Harding and colleagues proposed a new model of ethical decision-making that combined student demographic and academic variables with moral reasoning to predict self-reported rates of cheating on college tests (Harding et al. 2012). In their model, with regards to predicting ethical behavior, Harding and colleagues show a relationship between ethical reasoning, moral obligation, intention and behavior in the effect of predictive variables such as past high school cheating, involvement in Pan-Hellenic groups, moral obligation, and perceived behavioral control on the ethical behavior of undergraduate engineering students (Harding et al. 2012). With regards to relevance to our proposed model we need to note here that Harding and colleagues discuss academic ethics, not professional ethics. The authors themselves in their 2009 SEED report acknowledged “the intellectual and practical distinctions between these two forms of ethics” and have also suggested “that helping students learn ethical decision-making skills and principles that apply to both contexts should be a vital part of any ethics education program” (Harding et al. 2009, p. 6).

A recent quasi-experimental field study done by May and Luth investigated the effectiveness of ethics education on engineering and science students’ positive psychological outcomes, such as perspective taking, moral efficacy, moral courage, and moral meaningfulness (May and Luth 2013). Along with other study findings, the authors advise that engineering and science educators can train students to behave ethically in their future professional practice, regardless of “adverse consequences” and also as part of building their “professional identity” in their respective disciplines (May and Luth 2013, p. 566). Most recent work suggests applying virtue ethics and positive psychology to develop a new conceptual framework for more effective ethics education (Han 2015). Han suggests two potential educational approaches—moral modeling and participation in actual moral activity in engineering ethics classes. We have not found any literature yet on the implementation and the educational outcome of this proposed model.

The literature reveals that engineering ethics courses have stressed skills acquisition rather than behavior change (Shuman et al. 2004). We support the view that exercising ethical judgment does not mean that students are ethically educated or likely to behave in an ethical manner (Thoma 1994; Harding et al. 2013). The focus of this paper is to propose a model that accounts for both ethical awareness and ethical behavior. Applying this model will allow educators to build confidence and trust in our students that they will not only be able to identify ethical issues but practice ethical behavior.

Motivation plays a critical role in predicting an individual’s ethical behavior but it is not easy to assess. Within the proposed model we focus on motivations underlying ethical awareness and ethical behavior. This model can be readily translated into classroom activities and assessments that will focus on helping students understand the nature and value of professional and ethical responsibility. The model proposed here was developed to predict both the ethical awareness of engineering students and their subsequent ethical behavior, and to also be used as a pedagogical tool for designing courses addressing engineering ethics learning objectives. We have integrated the theory of planned behavior (Ajzen 1985), spheres of perceived control (Paulhus and Van Seals 1990), and person orientation (Graziano et al. 2012) into a single model. We also hope to offer, in our model various pedagogies and assessments to help engineering educators convey to the students the differences between academic ethics and professional ethics, as the work they are asked to complete in the particular course is directly applicable to their professional goals.

## Theory of Planned Behavior

**Fig. 1:**
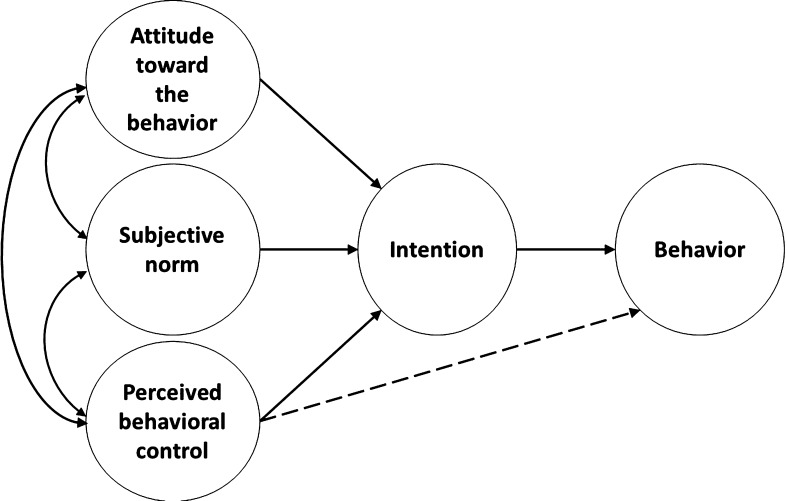
The theory of planned behavior. Adapted from Ajzen (1991)

We propose applying a well-established model of human behavior, Ajzen’s theory of planned behavior (Ajzen 1985), as a theoretical framework for understanding and predicting ethics in engineering and for informing engineering ethics pedagogy. Ethical decisions are complex, so it is important to understand what factors influence ethical awareness and subsequent decisions to behave ethically, and to also consider the impact of student motivations on both of these processes. The theory of planned behavior posits that an individual’s attitude toward the particular behavior, support from important others to perform the behavior (subjective norms), and their perceived behavioral control over performing the behavior strongly predict behavioral intentions; in this case intentions to act in an ethical manner. Intentions to act ethically will, in turn, predict ethical behavior (see Fig. 1). In this section of the paper we briefly outline the elements of the theory of planned behavior and how they relate to understanding engineering ethics and predicting ethical behavior. We will review the utility of the theory of planned behavior to predict ethical intentions and behaviors across a number of domains. We will then propose a revised model that can be used to predict ethical awareness and behavior as well as to teach engineering ethics.

### Attitude Toward the Behavior

Attitudes are the degree to which an individual has a positive or negative evaluation of a particular behavior. “Would [performing a specific ethical behavior] make me feel good or bad?” “Would it be wise or unwise?” “Would it be pleasant or unpleasant?” “Would it be fun or boring?” Different beliefs about a particular behavior contribute to the development of an overall positive or negative attitude towards performing the behavior (Ajzen 1985). Positive attitudes toward the behavior should increase an individual’s intentions to perform that specific behavior.

### Subjective Norms

These are individuals’ beliefs about whether people they care about would support them performing a behavior. For example, “People who are important to me think that I should [perform this ethical behavior].” Subjective norms are a function of beliefs about how supportive important others are for performing the behavior, and the individuals’ motivation to comply with these important others. Strong subjective norms in favor of performing the behavior should predict individuals’ intentions to perform that behavior; weak subjective norms should lessen behavioral intentions.

### Perceived Behavioral Control

This is the amount of control an individual feels they have over a particular situation or behavior. For example, “How easy or difficult will it be to [perform the ethical behavior] if I want to?” “If it were up to me, to what extent do I feel that I can [perform the ethical behavior]?” It is important to note the theory of planned behavior accounts for individuals’ perceived behavioral control, rather than their actual level of control over performing the behavior. Actual behavioral control is an individual’s opportunities and resources to perform the behavior and, depending upon the situation, perceived behavioral control may or may not reflect an individual’s actual behavioral control. Perceived behavioral control is a direct antecedent to behavioral intention, however, perceived behavioral control may also exert a direct effect on behavior in instances where perceived behavioral control is highly correlated with actual behavioral control (see Fig. 1) (Beck and Ajzen 1991). Overall, an individual’s level of perceived control should positively predict behavioral intentions and, in some instances, actual behavior.

### Behavioral Intentions

Central to the theory of planned behavior is the notion of behavioral intentions. Intentions are the motivational factors that influence how hard an individual is willing to try to perform the behavior and how much effort they will put into performing it (Ajzen 1985; Beck and Ajzen 1991). For example, “How likely is it that I will [perform the ethical behavior] the next time I suspect a breach of ethics?” In the theory of planned behavior model, behavioral intentions are the direct antecedents of behavior, and should be strongly and positively correlated to actually performing the behavior.

### Predictive Power of the Theory of Planned Behavior

The theory of planned behavior has been applied to predict a variety of behaviors such as smoking cessation, condom use, healthy behavioral choices, and pro-environmental behavior to name just a few. The theory of planned behavior has also been supported by decades of empirical research. In a typical study, participants complete validated measures of their attitude toward the behavior in question, the subjective norms supporting them performing the behavior, their perceptions of the degree to which performing the behavior is under their control, and their intentions to perform the behavior in the near future. These studies typically report a measure of the degree to which attitudes, subjective norms, and perceived behavioral control predict behavioral intentions. Studies where actual behavior is also measured also report the predictive power of intentions on subsequent behavior. These metrics vary, so we will use the “percent of variance accounted for” as our measure of the predictive power of the variables in the theory of planned behavior to predict behavioral intentions and actual behavior. A robust predictive model should predict a significant amount of the variance of a given behavior or outcome (in our case ethical intentions or behaviors) with a small number of predictors. No psychological model with reasonable parsimony will ever predict 100 % of a given intention or behavior, but the theory of planned behavior has proven to be a robust predictor of intentions and behavior across many domains.

Meta-analyses of studies using the theory of planned behavior indicate that a person’s attitudes, subjective norms, and perceived behavioral control typically account for about 40 % of the variance in his or her behavioral intentions and 30 % of the variance in his or her subsequent behavior (see Armitage 2001). For example, a study of smokers trying to kick the habit revealed that a cigarette smoker’s attitude toward not smoking, subjective norms supporting not smoking, and perceived control over not smoking predicted 49 % of the variance in intentions to quit smoking (Norman et al. 1999). Perceived behavioral control was the strongest predictor of intentions to quit. Applying the theory to a different behavior, the attitudes toward regular exercise, subjective norms supporting participation in regular exercise, and perceived behavioral control over exercising regularly of gymnasium members accounted for 49 % of the variance in intentions to exercise regularly (Armitage 2005). Perceived behavioral control and subjective norms were the strongest predictor of intentions to participate in regular exercise. Intentions to exercise regularly and perceived control over regularly exercising accounted for 22 % of the variance in the average number of gymnasium visits across 12 weeks. In a study of the environmental behaviors of over 200 households revealed that attitudes to recycling, subjective norms supporting recycling, and perceived behavioral control over recycling accounted for 26 % of the variance in intentions to participate in a household recycling program (Tonglet et al. 2004). In this study, attitudes toward recycling were the strongest predictor of recycling intentions.

These examples illustrate the variety of behaviors the theory of planned behavior has been used to understand and predict. Admittedly, the examples above involve fairly uncontroversial behaviors where identifying the “right” behavioral course of action (not smoking, going to the gym, and participating in household recycling) is clear. The theory of planned behavior has also been applied to understanding the determinants of ethical behavior. Identifying the right course of action in response to an ethical dilemma is often not straightforward. In addition, acting ethically or refusing to act unethically may not always be completely under an individual’s volitional control. In the next section we review research that has applied the theory of planned behavior to understanding and predicting ethical behaviors.

### Predicting Ethical Intentions and Behavior

The theory of planned behavior has been used previously to predict ethical intentions and behaviors across a variety of different professional domains such as accounting, medicine, and information technology. In the domain of Engineering Education, Harding and colleagues, as discussed earlier in the paper, have been using the theory of planned behavior to predict students’ academic ethics intentions and behavior (e.g. college cheating). With the exception of their work, research in the domains of accounting, medicine, and information technology, typically utilizes case studies, similar to those used in the instruction of engineering ethics. Research participants read a series of vignettes or scenarios depicting ethical dilemmas and are asked to report their intentions to act ethically, or to refuse to act unethically. A relevant example is a study of ethical behavior in the medical field, where nurses were shown scenarios that depicted inadequate patient care and asked if they would report the person responsible for the situation. One scenario states:

You observe that a doctor in the intensive care unit made a fairly minor error in patient care. The patient is stable and appears in no immediate danger. Whereas the physician generally has a good reputation, you have seen a series of past mistakes by this physician and believe the doctor to be incompetent. You believe that the doctor has no idea that an error has been made. You personally like this doctor and think the doctors may be overworked. The doctor is also very close friends with your supervisor. You must decide whether or not to report the doctor to your supervisor. (Randall and Gibson 1991, p. 114)

No actual behavior was measured in this particular study, but attitude toward the behavior, subjective norms, and perceived behavioral control accounted for 61 % of the variance in nurses’ intention to report the person responsible for the poor patient care. Interestingly, while attitudes toward the behavior and social norms were a statistically significant predictor of nurses’ intentions, perceived behavioral control was not (Randall and Gibson 1991).

In a second example, researchers conducted a study in response to a series of high-profile breaches of ethics in public accountancy firms. Professionals from public accounting firms responded to vignettes depicting ethical dilemmas faced in public accounting. Attitudes and subjective norms combined to predict 41 % of the variance in intention to behave ethically. Again, perceived behavioral control failed to be a significant predictor of intentions to behave ethically (Buchan 2005). With the exception of the work of Harding et al. who have used the theory of planned behavior to study the intentions of engineering students to cheat—arguably an ethical dilemma, no studies that we are aware of have applied the theory of planned behavior to study behavior in response to ethical dilemmas in engineering.

### Moral Obligation

While the theory of planned behavior as it was originally conceived has shown promise as a framework for predicting responses to ethical dilemmas, Ajzen (1991) noted that in situations involving moral or ethical behavior, an individuals’ personal feelings of moral obligation or responsibility to perform, or decline to perform, behaviors should also significantly influence behavioral intentions. Many studies using the theory of planned behavior to predict ethical decision-making have taken heed and added individuals’ perceived moral obligations to their model. Perceived moral obligation is typically measured by items such as “I would not feel guilty if I [performed the unethical behavior in question]”, “[The unethical behavior in question] goes against my principles.” The inclusion of moral obligation has significantly improved the prediction of behavioral intention in studies of behaviors that include a moral or ethical dimension such as cheating and lying (Beck and Ajzen 1991), driving violations (Parker et al. 1992), and shoplifting (Tonglet 2000, 2002).

An excellent example is Beck and Ajzen’s (1991) study of three unethical behaviors: cheating on an exam, shoplifting, and lying to get out of a class assignment. The addition of a measure of moral obligation to attitudes, subjective norms, and perceived behavioral control increased the prediction of intentions to perform these dishonest behaviors from 66 to 72 % of the variance. The addition of moral obligation also improved the power of the model to predict self-reported behavior from 27 % of the total variance to 40 %. A study using engineering students as participants replicated these effects and found that attitudes, subjective norms, and perceived behavioral control were significant predictors of intentions to cheat on a test during the following semester. However, moral obligation was the strongest predictor of ethical intentions (Harding et al. 2012).

Although there has been some debate about the efficacy of including a measure of moral obligation to predict intentions and behavior in morally relevant situations (see Gorsuch and Ortberg 1983), the weight of evidence suggests that a model will yield better predictive power if moral obligations are considered. Given our focus on predicting and teaching ethical behavior in engineering, the benefits of including moral obligations in engineering ethics curriculum must also be considered. In the next section we propose a new, two-part model as a theoretically and empirically grounded framework for predicting and teaching engineering ethics.

## A New Model to Teach Engineering Ethics and Ethical Behavior

**Fig. 2:**
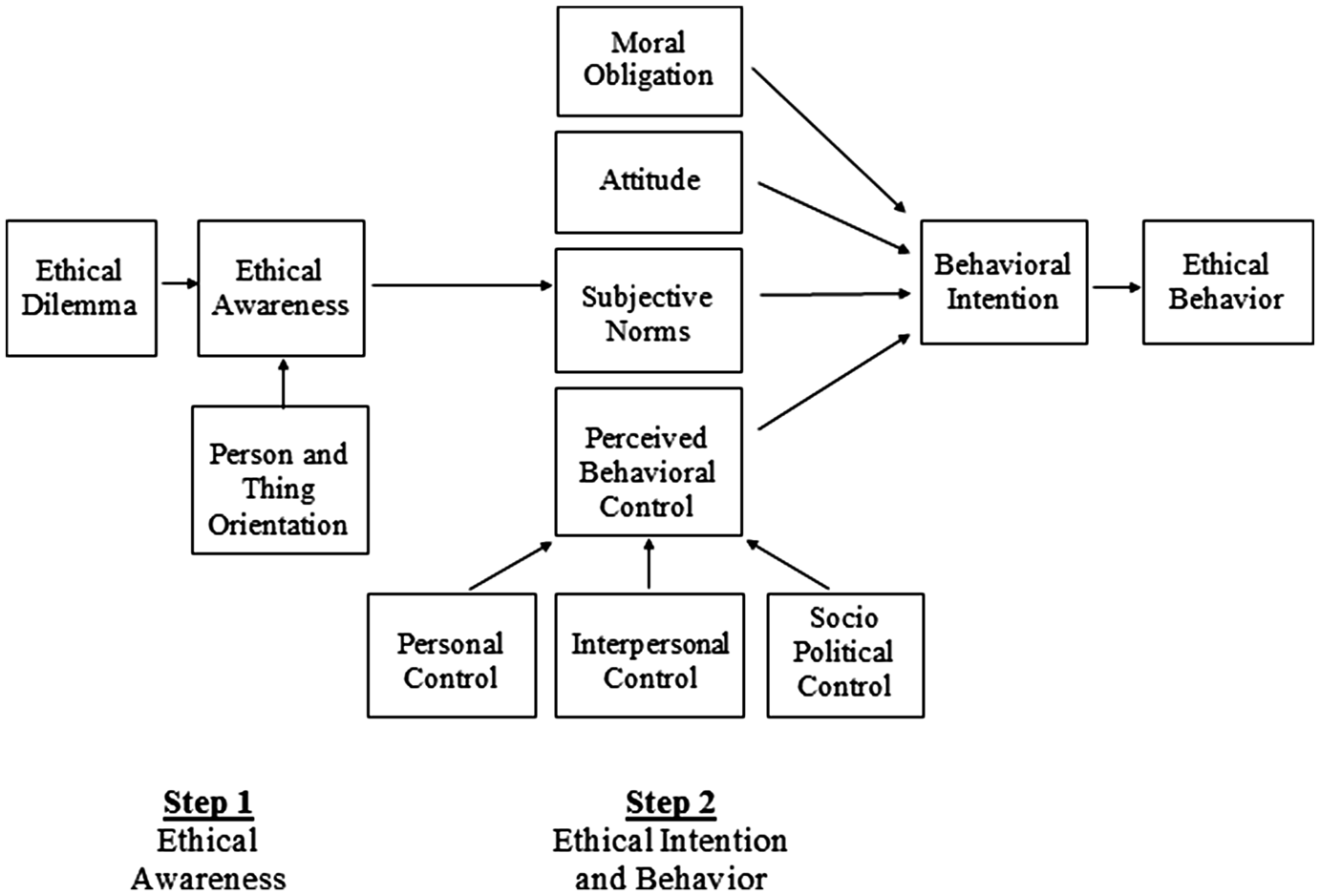
New model to predict ethical awareness and ethical behavior

We propose a new model based on the theory of planned behavior that will help measure engineers’ ethical awareness and predict subsequent behavior, that can also be used as a tool to refine engineering ethics pedagogy. We hope we have convinced the reader of the need to improve the curriculum for the instruction of engineering ethics and to further empirical research into the process by which engineers make ethical decisions, and how this may translate into ethical behavior. Using the theory of planned behavior as our framework we propose an extension of the original model to include: moral obligations, a more nuanced approach to perceived behavioral control, and the consideration of ethical behavior as a two-part process whereby ethical awareness precedes ethical intentions and behavior (see Fig. 2).

### Inclusion of Moral Obligation

The first point of departure from the traditional theory of planned behavior model is the inclusion of moral obligation. As discussed previously, we are not the first researchers to include moral obligation in a model to predict ethical behavior (for example see Harding et al. 2007), but we feel a model that includes attitudes, subjective norms, perceived behavioral control, and moral obligations offers greater predictive power than traditional theory of planned behavior models for ethical behaviors. The inclusion of moral obligations also offers opportunities for a more integrative pedagogical approach. Curricula may include discussions of students’ personal moral obligations and feelings across a variety of ethically ambiguous situations. It can also take into consideration cultural differences and similarities in moral obligations, and students prior ethical behavior.

### A Hierarchical Approach to Perceived Behavioral Control

The relative importance or predictive power of attitudes, subjective norms, and perceived behavioral control has been shown to vary across different behaviors (Beck and Ajzen 1991). An individual’s perceived behavioral control is a strong predictor of his or her behavior in domains that do not involve ethical dilemmas, yet contributes less than optimal predictive power for ethical behaviors. In ethical dilemmas, attitudes and social norms consistently predict the majority of the variance. We argue this may be a case of a lack of correspondence between the measure of perceived control and the level or sphere of behavioral control that the ethical dilemma at hand resides in. Our new model addresses this issue by considering three different spheres of perceived behavioral control: personal efficacy, interpersonal control, and socio-political control (Paulhus 1983).

Ajzen (1991) points out that behavioral intentions can only predict behavior to the extent that the given behavior is under volitional control—the extent to which an individual can decide at will if they will perform or not perform a particular behavior. We argue that an engineer’s volitional control to act ethically or refuse to act unethically varies greatly from situation to situation, and that many ethical dilemmas engineers face are predominantly outside his or her direct control. Consider the following scenarios:

- Scenario #1: A materials engineer has taken over the last part of a government project with a Research and Development firm, with an impending deadline and a fixed budget. The materials engineer is responsible for completing the project by making the last call on the implementation of a material in a biomedical device. She faces a dilemma of using a polymer, available in the lab but with testing specifications acknowledging the possibility of a low minimal risk of damaging human skin when in contact with one of the already used materials in the device. The other option the engineer has is to select a newly developed polymer with no known risks but with a significantly higher price than the other material and a long delivery time.
- Scenario #2: A mechanical engineer who does CAD design for a semi-conductor company is asked by one of the company’s electrical engineers to change the company information on the schematics of a competitor’s component, which they are using in one of their products. The electrical engineer has received a call from the company CEO asking for the change to be made so she can send the schematic to the client.
- Scenario #3: A software engineer works for a government healthcare agency and is constantly asked by the leadership of the agency to apply temporary and inefficient patches to problems in their software, rather than spend the necessary time to properly correct the problems with the code. This behavior costs the agency hundreds of thousands of dollars each year and the software engineer suspects foul play.

Engineers face situations such as theft, inaccurate reporting of numbers or specifications, or other misconduct such as scenario #1 whereby acting ethically or refusing to act unethically may be almost completely, if not completely, under the individual engineer’s volition. However, many situations engineers face require more than their personal motivation and volition to behave ethically. These situations may require the cooperation of others to behave in an ethical manner such as scenario #2 depicted. Engineers may also face ethical dilemmas that are embedded within a context of unethical behavior that runs through an entire project team, organization, or agency—scenario #3 for example. We argue that these ethical dilemmas are qualitatively different with regard to an individual engineer’s personal volitional control; but regardless once the engineer is aware of an ethical dilemma or breech, he or she must enact some behavioral response (action or inaction). When the situation affords, the individual engineer’s complete control of their behavior, his or her attitudes, subjective norms, and moral obligations should be strong predictors of ethical intentions and, in turn, behavior. However, when faced with a situation where the engineer does not have complete control over his or her’ behavioral response, it is important to consider perceived behavioral control. We argue that considering the different spheres of perceived control will increase the precision of prior measures of perceived behavioral control in application of the theory of planned behavior. Students in regards to engineering ethics will become aware of their personal volition in situations in each sphere and be better equipped to respond to ethical dilemmas.

### Spheres of Control

**Fig. 3:**
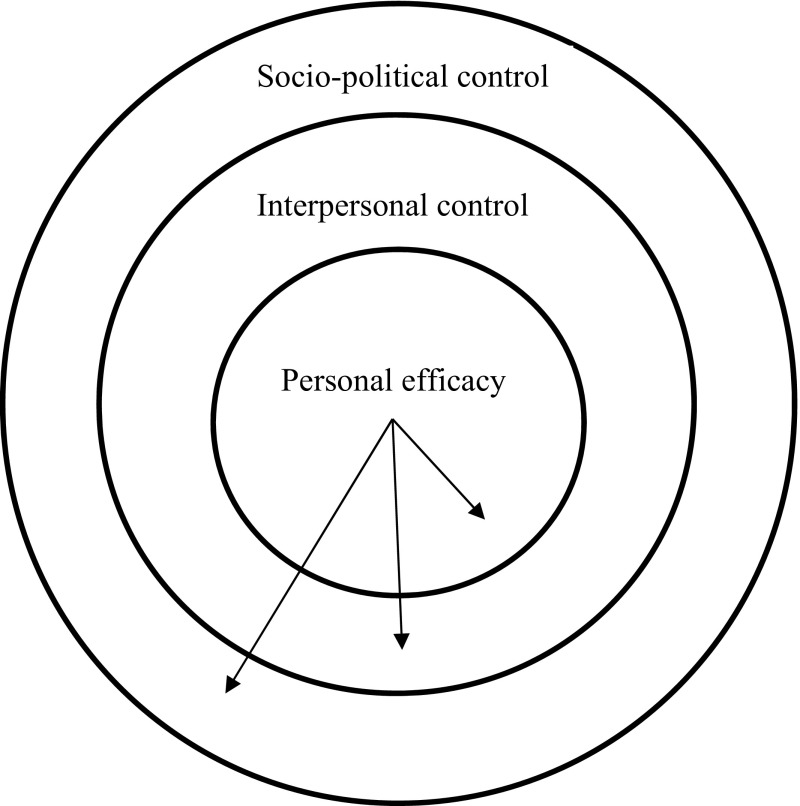
Spheres of control. Adapted from Paulhus (1983)

Spheres of control reflect the degree to which people feel they have agency or control over specific areas of their lives. Paulhus and Van Seals (1990) describe three distinct spheres or domains for control: personal efficacy, interpersonal control, and socio-political control (see Fig. 3). People differ in the degree to which they perceive they are able to exercise control in different domains. The domain closest to the core self is personal efficacy (self-achievement). This domain is concerned with the motivation to have mastery or control over objects, events and tasks. When someone has a mechanical problem for example, they may want to have control over how they fix it, which gives them satisfaction. This is the domain where ethical behavior such as theft or misrepresentation of one’s work resides. The next sphere is interpersonal control. This sphere is concerned with regulating and controlling relationships with other people in dyads and groups. For example, “Can I defend my position in a team meeting?” “Can I influence my team to act ethically?” The outermost layer is socio-political control. This is the domain concerned with controlling institutions and organizations. Individuals who score high in socio-political control are most likely to stand up and oppose an organization they feel is behaving unethically. A strength of the proposed model is the more nuanced approach to perceived behavioral control than has been presented in previous applications of the theory of planned behavior to engineering ethics. We argue that measuring students’ perceptions of behavioral control at each sphere and including instruction of the potential ethical dilemmas in each sphere highlighting ways of effectively and ethically responding to them will enhance both ethical awareness and potential future behavior. The spheres of control engineers and engineering students score highly in should predict their perceived behavioral control to act ethically, or not to act unethically, when faced with dilemmas in those domains. Identifying ethical dilemmas in each sphere and being instructed with strategies to effectively deal with them are important considerations for teaching engineering ethics.

### Ethical Awareness

The last part of our new model considers the process underlying students’ ethical awareness: their ability to identify a breach of ethics in a potentially ambiguous situation. Ethical decisions, particularly those faced by practicing engineers, are often complex and may involve the behavior and intentions of others. We extend the theory of planned behavior to account for two distinct processes in ethical behavior: (1) the recognition of a breach of ethics which we term “ethical awareness”, and (2) engaging in ethical behavior. Our extension of the theory of the planned behavior model to this point accounts for the second part of this process: ethical behavior. We propose a two-step model of ethical behavior (see Fig. 2). The first step is the awareness of a breach, or potential breach, in ethics. Ethical awareness is the degree to which, given certain information, an individual detects an ethical problem or the potential for an ethical problem to occur. Scenarios or vignettes depicting real or potential breaches of ethics are widely used to teach professional ethics to engineering students. Students identify the correct or ethical response to such simulations. An example from our own research with engineering students is “You are on a proposal writing team. In the orientation briefing, the head of the team provides the following guidance: ‘We really have to win this one. I want you to be really optimistic in what you write.’ How do you interpret her advice?” (Ethical vignette from Lockheed Martin Ethics Challenge Game). The awareness of a breach, or potential breach, in ethics is an important antecedent to selecting the most ethical course of action. Ethical dilemmas, both real and simulated, run the gambit from clear to ambiguous. Situations where behavior is ambiguous leave the detection of a possible ethical breach in question. We argue individual differences in orientation to the social environment may help determine how individuals detect ethical breaches in ambiguous situations.

### Person and Thing Orientations

Individual differences can serve as powerful motivators for behavior. The scenarios presented above make it clear that ethical awareness and subsequent ethical behavior are predicated on interpersonal processes. We argue individuals’ sensitivity and attention to the people and interpersonal processes in their environments will significantly predict their ability to detect breaches in ethics. People selectively orient to the people and things in their environment; they either strongly or weakly orient to the people or physical objects around them. Consider your interest in the following: “Stopping to watch a machine working on the street” or “Making the first attempt to meet a new neighbor.” Are you interested in one more than the other or both? People who are strongly thing oriented respond positively to scenarios concerning working or modeling a machine. People who are strongly person oriented respond to situations involving interpersonal interactions. Once considered opposing ends of a continuum, recent research indicates that person and thing orientations are separate constructs: such that individuals can embody different person and thing orientation configurations or specializations (Graziano et al. 2012; Little 1986; Tay et al. 2011; Woodcock et al. 2013). Individuals who score high on thing orientation have been classified as “thing specialists.” Those who score high on person orientation have been classified as “person specialists.” Those who score high on both person and thing orientations are classified as “generalists,” and those low on both are classified as “non-specialists” (Little 1986).

**Fig. 4:**
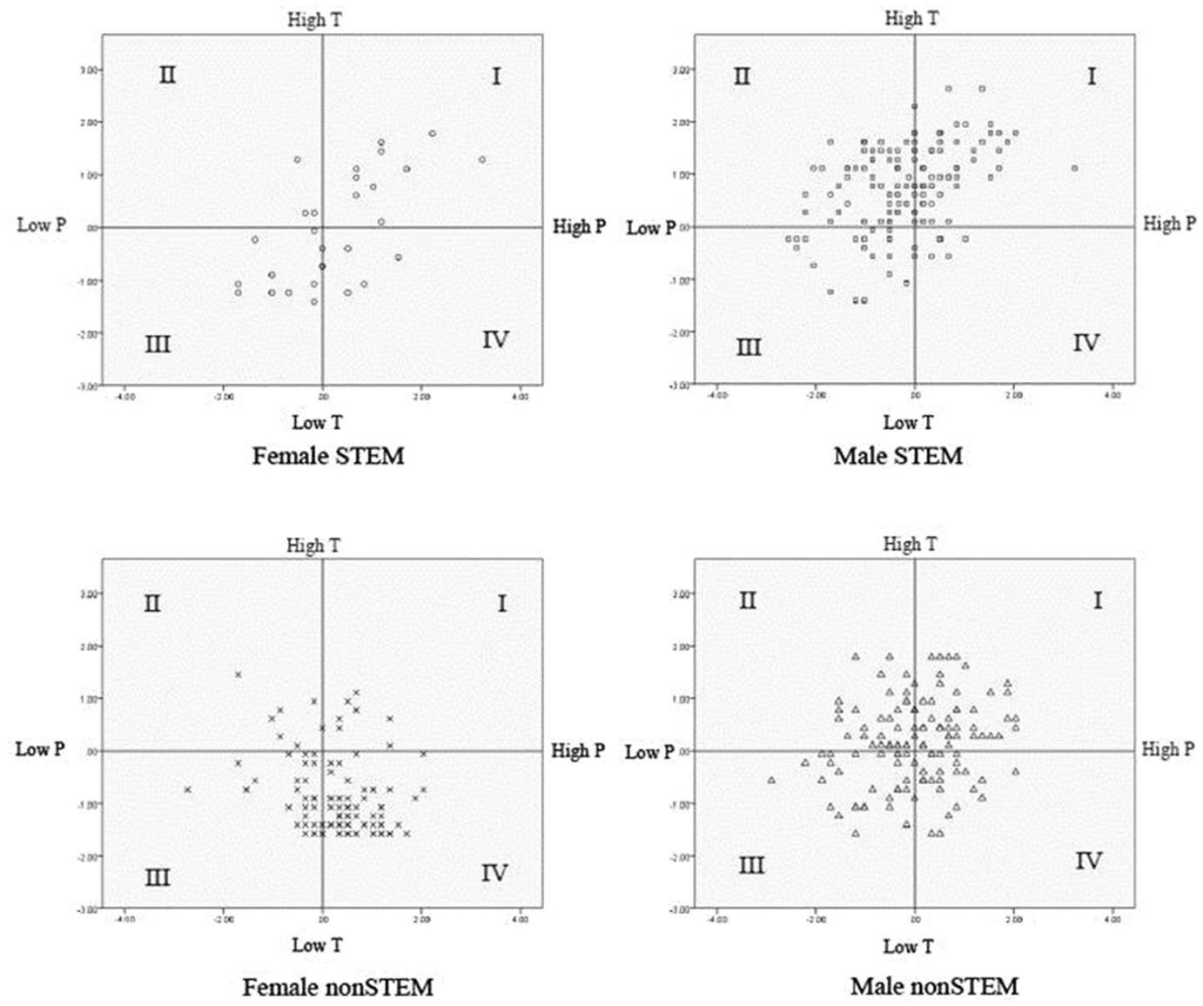
Person and thing orientation configurations of US science, technology, engineering, and mathematics (STEM) and non-STEM majors by sex. **I** Generalists, **II** thing specialists, **III** non-specialists, **IV** person specialists *Source*: Graziano et al. (2012)

The extent to which people are interested in the people and things in their environment is strongly associated with academic and vocational choice (Graziano et al. 2012; Su and Rounds 2015). Not surprisingly, engineers and engineering students typically score high on measures of thing orientation; however they also frequently score high on measures of person orientation (Woodcock et al. 2013). Thus, engineers and engineering students are often “thing” specialists or generalists. Figure 4 depicts the personal and thing orientation configurations of U.S. science, technology, engineering, and mathematics (STEM) majors. Both, male and female STEM majors typically report high thing orientation scores as would be expected. However, many of them also report high scores on person orientation—but their levels of person orientation are quite variable. This is an important point, because detecting ethical dilemmas involves an understanding of the motives and intentions of other people. The degree to which engineers-in-training orient to the people and interpersonal interactions in their environment should be predictive of their ethical awareness. Engineers with strong person orientations should be better able to detect ethical dilemmas than those with weaker person orientations. Understanding students’ levels of person and thing orientations (and students’ understanding of their own) may also be critical for the detection of an ethical breech where ethical awareness is predicated on the relative impact of the dilemma on people and their welfare, or “things” such as process and materials. Ethical dilemmas such as those presented in our three scenarios have both “person” and “thing” components that may be detected and weighted differently according to individual engineers own configuration of person and thing orientations. For example, in scenario #1, the dilemma resides in the tension between the properties of the polymers and adherence to budget and schedule—“thing” components, and the potential risk to the welfare of those who come in contact with the polymer—a “people” component. Differentially weighting the importance of the material versus the human costs has implications for an engineer’s course of action. We believe that measuring and discussing person and thing orientations as they pertain to ethical dilemmas is an important pedagogical approach to help students to gain insight into their own orientations and how they approach ethical dilemmas, and how they might approach them more effectively.

Therefore, we propose to include person and thing orientations as predictors of ethical awareness in our model and to also use these orientations as a pedagogical tool.

It is important to note that engineering students arrive in the classroom with varying levels of prior ethical behavior. Prior behavior is a potent predictor of future behavior, and must be taken into consideration, particularly when conducting research on the predictive power of the components of our model on behavioral intentions and actual behavior. The accurate measurement of prior behavior, particularly ethical behavior, is challenging as it often relies on retrospective accounts that are subject to any number of cognitive biases and impression management concerns.

## Implications for Pedagogy

The theory of planned behavior has been used as an effective framework for understanding determinants of ethical behavior. We argue our new model will provide the following: a useful framework for teaching engineering ethics; address recommendations from the Colby and Sullivan study to define ethics and professional responsibility broadly; and use active pedagogy by integrating ethics with the specific course learning objectives. Keefer and colleagues stress on the importance of aligning classroom-based assessments to clear ethical learning objectives (Keefer et al. 2014). This alignment, they suggest will help both students and instructors to reach those objectives. We suggest the learning objectives are designed specifically around Fink’s approach “A Self-Directed Guide to Designing Courses for Significant Learning”. Fink’s approach is an integrated course design based on first developing individual components such as identifying situational factors and learning goals, next integrating the individual components into an overall structured course, and finally planning for student factors such as grading system and course syllabus (Fink 2003). Fink offers taxonomy of significant learning that has a hierarchy, which is meaningful in this design context such that knowledge, application, and integration are defined. Our model aligns with Fink’s guidelines for course design as it provides a framework for clear outcomes learners must achieve: positive attitudes toward ethical behavior; a feeling of support to act ethically; a sense of moral obligation; awareness of spheres of control and how they relate to implementing ethical behavior. These student outcomes can be easily assessed at the beginning of a course and evaluated throughout. The model also allows for the selection of specific instructional resources and learning activities that tie directly to each student outcome.

We suggest concepts used with our new model need to be in alignment with learning objectives where students will be able to (1) recognize ethical issues; (2) exercise ethical awareness; (3) apply ethical behavior. The first objective aligns with Fink’s Fundamental Knowledge. This objective will first, help students to gain understanding of the legal, professional, historical, and personal definitions of engineering ethics; and second, it will help students to be familiar with some distinctions among ethical concepts, such as moral dilemma, responsibility, whistle blowing, professional ethics versus personal ethics, utilitarianism, duty, virtue and rights. The inclusion of the different spheres of control will help students more accurately assess an ethical dilemma with respect to their level of personal volition to act. The second objective falls into Fink’s Integration, Human Dimensions, and Caring goals as it helps students learn contemporary and historical legal, professional, and personal reasons why an engineer should be ethical, and also helps students identify ethical dilemmas and challenges them to exercise ethical awareness. Our model will also inform students about their relative levels of person and thing orientations and how this might influence their consideration of the material and personal aspects of an ethical dilemma.

**Fig. 5:**
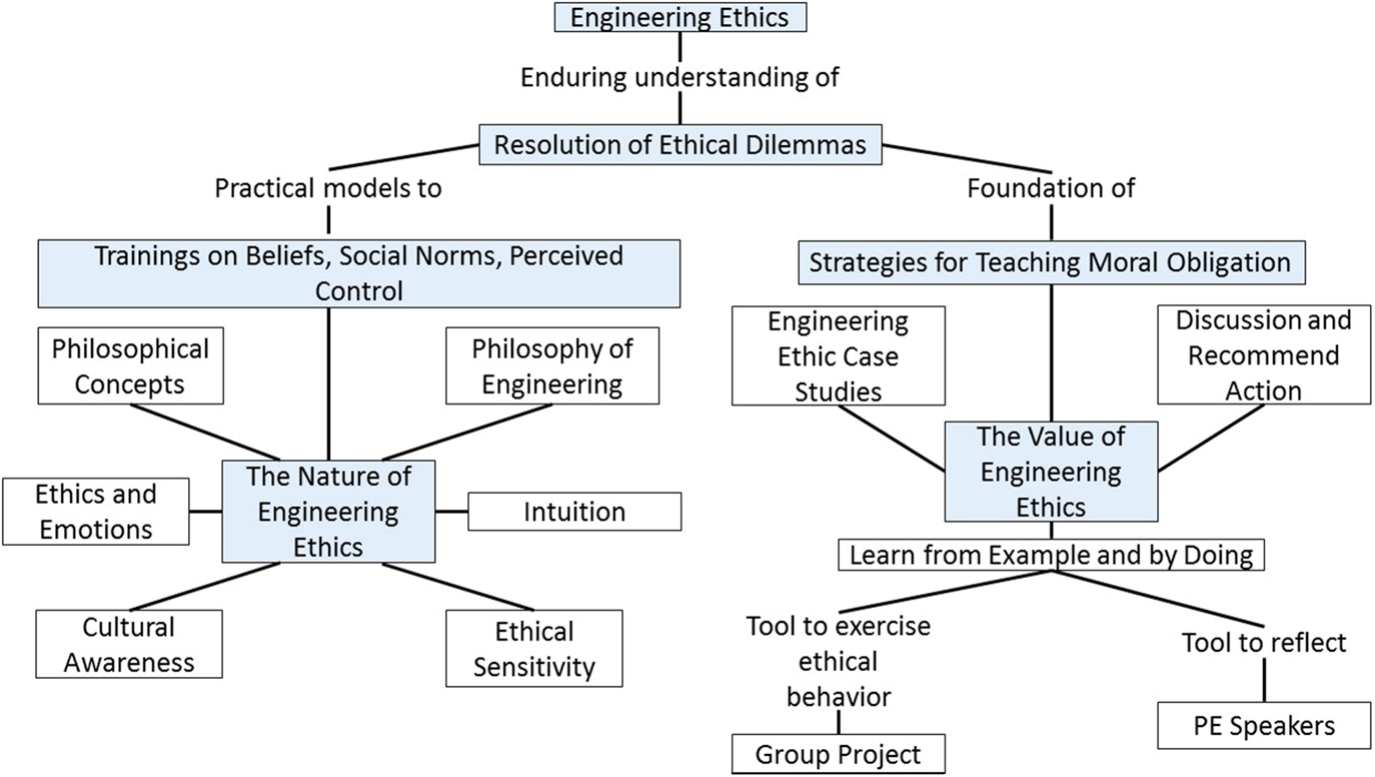
Concept map illustrating the connections between the basic concepts

The third objective aligns with Fink’s Application, Integration, and Learning. This objective helps students foster the development of logical skills in ethics by first, using common ethical dilemmas to identify possible actions; second, to identify possible consequences of those actions; and lastly, to practice ethical behavior. In addition, this learning objective stimulates students’ ethical behavior and promotes a sense of responsibility. These three ethics focused learning objectives will be integrated with other learning goals of the specific course. The concept map (Fig. 5) illustrates the connections between the basic concepts in the course. The course can be found at top center of the concept map in the large block. The three fundamental concepts, arrayed below the course block, serve to help students learn the nature and the value of Engineering Ethics and to develop ethical awareness and behavior for resolution of ethical dilemmas.

One cannot expect that students will be able to understand and adapt to professional standards and values of the community simply by remotely and abstractly learning and observing the behavior of professionals. Learning by observation requires interpretation. The rationale and assumptions that underlie behavior are not always obvious. Observational learning, alone, is not an efficient method of communicating critical issues. Based on these arguments the proposed pedagogical model will focus on training engineering students to resolve ethical dilemmas based on (1) learning about moral obligation and (2) training positive attitudes towards ethics, social norms, and perceived control.

### Teaching Moral Obligations

The nature of engineering ethics in our model is viewed through the lens of presenting engineering as human-centered discipline. The pedagogical strategies to teach moral obligation with a core focus on the nature of engineering ethics will be accomplished by the integration of active and cooperative learning, debate, and reflection. Philosophical concepts and philosophy of engineering, specifically, will be introduced to help students understand the nature of engineering ethics. Additional concepts, such as culture, ethics and emotions, ethical sensitivity, and intuition will be discussed to address the diverse background students have, while also helping them build on their ethical awareness as a foundation to resolve ethical dilemmas they may encounter (see C-map, left part). The pedagogical strategies utilized to teach moral obligation will provide an active and interactive learning environment. Furthermore, the presented concept will be meaningful and useful to all students by accounting for the different ways students learn.

To give students a more realistic understanding of their moral obligation and relationship to the real world, as well as minimizing the nature and limitations of the case studies approach, our proposed model provide opportunity for dialogue as a form of active learning. Dialogue can be used as a way to give students experience “engaging in conducting comprehensive research into the topic, gathering supporting evidence, collaborative learning, delegation of tasks, improving communication skills, and developing leadership and team-skills all at one go” (Christudason 2003). Students can present their argument maps, discuss it with others and play the role of being an expert briefing a committee. This activity will help students improve their skills of critical thinking, collaboration, and problem solving (integration with other course specific learning goals). Additionally, engaging in dialogues will help train students in developing skills for the resolution of ethical issues, along with preparing future engineers to exercise ethical behavior.

### Teaching Attitudes Towards Ethical Behavior, Social Norms, Perceived Behavioral Control and Intentions in Ethical Behavior

**Fig. 6:**
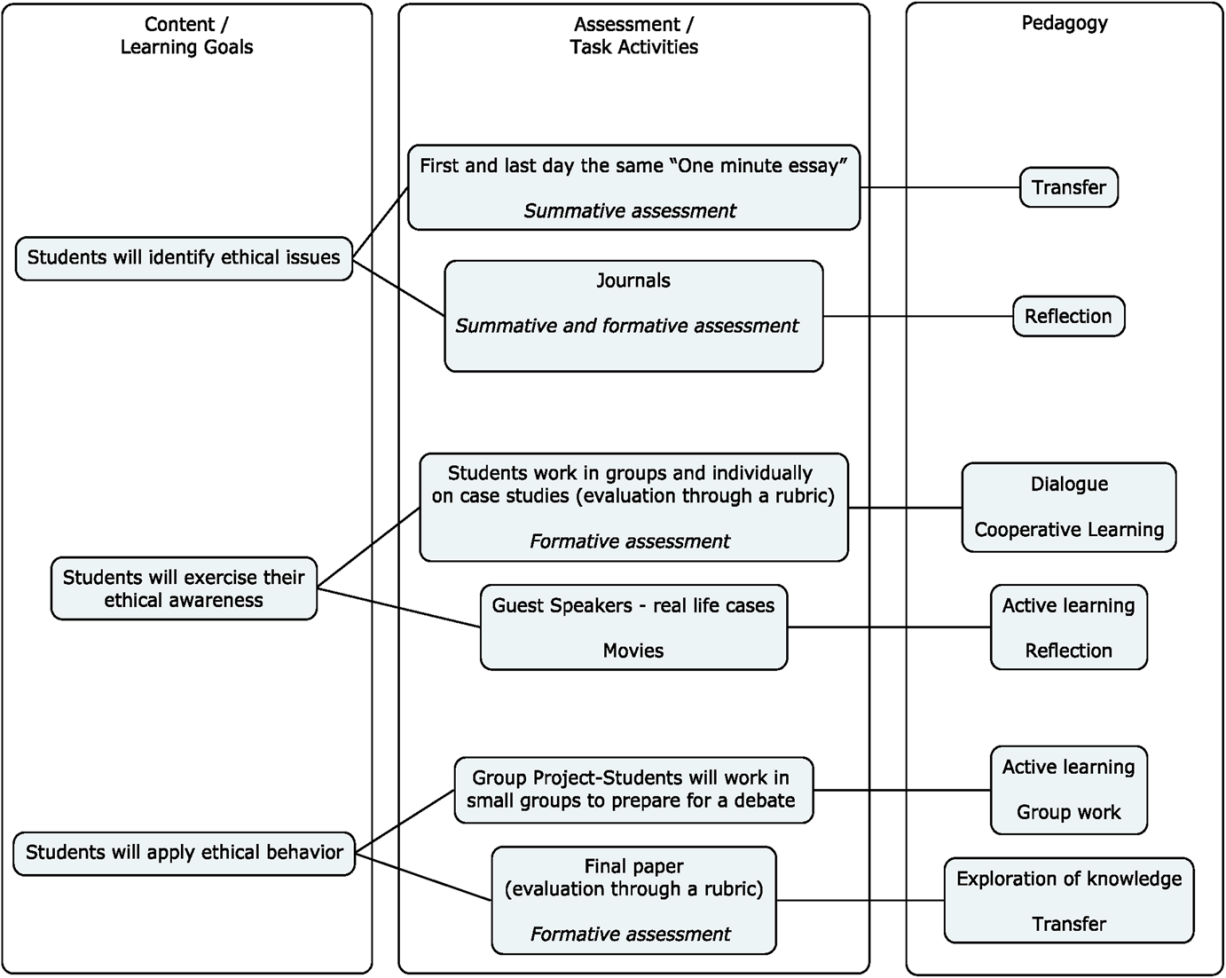
Alignment between the content, assessment, and pedagogy. *Source*: Bairaktarova and Evangelou (2011)

We argue ethical awareness alone is not sufficient to predict ethical behavior. In our model, pedagogical strategies are incorporated for training positive attitudes, social norms, moral obligation, and perceived controls. The pedagogical strategies to train student’s attitudes, social norms, perceived behavior control and intentions with a core focus on the value of engineering ethics will be accomplished through active learning, group work, reflection, exploration of knowledge, and transfer. The success of an interactive learning classroom depends on students’ contributions and engagement. In our model, besides a lecture about the nature and value of engineering ethics, listed in the content section of the course design, and social norms, two different forms can be introduced: class discussion and group work. The discussion approach to instruction, commonly known as discussion teaching, is a pedagogical method that has active learning of students and instructors at its core (Christensen 1991). Discussion teaching is organized to (1) create shared responsibility for teaching and learning; (2) honor the voices, experiences, and worldviews of students; (3) promote democratic participation in the teaching/learning dynamics; (4) develop critical thinking and problem solving skills; and (5) create a community of learners who work together in the pursuit of knowledge (Brookfield and Preskill 2005; Dillon 1994; Shor and Freire 1987). Discussions about readings can introduce students to important dimensions of beliefs and social norms. To prepare these discussions, students have to read the required texts to answer questions that encourage focusing on the readings’ central points. Students’ jobs are to read the texts, answer all the assigned questions in about one page, and submit it to the class. The idea is that students are best prepared for class discussions when they have already thought about the ideas that will be discussed in class at home. Examples such as reflective practice through personal journals, reflection after guest speakers, reflection after watching a movie, could be utilized using the proposed model (Fig. 6, Content, Assessment, and Pedagogy).

The purpose of reflection is to reinforce what students are learning through explicitly thinking about the assigned content (Bransford et al. 2000; Schon 1983; Svinicki 2004). One way of encouraging reflection is for the instructor to challenge students to explain what their thoughts are after these interactions. Another pedagogical approach in the form of transfer can be applied to teach students attitudes towards ethical behavior, social norms, perceived behavioral control and intentions in ethical behavior. According to Svinicki (2004) the ability to use knowledge in multiple contexts is evidence of true understanding. This idea is known as transfer. By providing students with progressively more philosophical concepts of the nature and value of engineering ethics, code of ethics, and ethics cases reflecting on the spheres of control, our model encourages the transfer and reinforcement of ethical awareness for resolution of ethical dilemmas from one context to another. Learning from experience through doing and guided by the instructor, students will be able to see how the knowledge they have gained in previous lectures and activities can be applied to resolving ethical dilemmas in cases and projects they currently work on outside of class.

### Understanding of the Spheres of Control that Ethical Dilemmas Can Operate At

As we have discussed ethical decisions in engineering can involve personal behavior (*I have a chance to fudge some numbers and get more $$. No*-*one will ever know. But will I act ethically?*), and/or potential influence of the behavior of others (*My team are getting pressure from management/the client to fudge the numbers. Can I influence my team/group to act ethically?*) and/or the behavior of entire organizations (*It is typical for my organization to inflate the numbers. Can I change this?*). Our model provides an opportunity for the strategic use of case studies at all levels of spheres of control. Perceived behavioral control should predict behavioral intentions (in concert with subjective norms and attitudes toward the behavior) and also predict actual behaviors. However, different spheres of control should predict different outcomes for different moral dilemmas.

Here we also need to consider that real ethical decisions can dramatically contradict moral choices made in hypothetical scenarios (FeldmanHall et al. 2012). While multiple choice questions are the format offered at the Fundamentals Exam, students must be prepared to face ethical dilemmas for which there is not always a straightforward answer and that includes diversity of moral codes as a result of diverse cultural, educational, professional backgrounds.

Understanding of how the spheres of control that ethical dilemmas can operate in could also further our understanding of how these differences affect the decision-making process under both real and hypothetical conditions (FeldmanHall et al. 2012).

### Understanding Students’ Differing Orientation to the People and Things in Their Environments

Person and thing orientations describe the degree to which individuals selectively orient to the people and objects in their environments. Person orientation should be predictive of knowledge of an ethical breach in an ambiguous situation. Engineering students are typically higher in thing orientation than person orientation, which may pose a challenge for ethics instructors. Pedagogy may need to be built around teaching students to be more attuned to the personal aspects of their environments. Teaching ethical awareness may benefit from understanding many that engineering students are highly thing-oriented and vary quite markedly in person orientation. How traditional ethical vignettes are presented should be considered in the design of the assessment and the pedagogy when teaching ethical awareness, for example, based on students’ motivation to engage with people or things in their environment the presentation of ethical vignettes could stress particularly on the facts or the events in the text.

Example vignette: “You are on a proposal-writing team. In the orientation briefing the head of the team provides the following guidance: ‘We really need to win this one. I want you to be really optimistic in what you write’” (Locked Marthin Ethics Chalende Games). Many cues that indicate potential ethical dilemmas or breaches may be non-verbal rather than verbal communication. This has implications for both teaching and measuring ethical awareness. Students are typically presented with written vignettes or scenarios depicting potential ethical dilemmas. The written presentation of salient aspects of the scenario may overestimate the degree to which students, particularly those lower in person orientation, will be able to detect ethical breaches in real life. We argue teaching ethical awareness to engineering students could benefit from: presenting video clips of potential ethical dilemmas, and leading discussions regarding verbal and non-verbal cues that indicate a potential breach of ethics. This activity may require instruction in interpreting interpersonal cues to detect ethical dilemmas.

## Final Remarks

Motivation plays a critical role in predicting an individual’s ethical behavior, but motivation is not easy to assess. Within the proposed model we focus on motivations underlying ethical awareness and ethical behavior. The model proposed here was developed to predict both the ethical awareness of engineering students and their subsequent ethical behavior, and to also be used as a pedagogical tool for designing courses addressing the engineering ethics learning objective. We have integrated the theory of planned behavior (Ajzen 1985), spheres of perceived control (Paulhus and Van Seals 1990), and person orientation (Graziano et al. 2012) into a single model.

It is vital to understand the decision-making process and the motivational variables that influence ethical decisions when creating pedagogy to effectively teach ethical awareness and ethical behavior. This model can be readily translated into classroom activities and assessments that will focus on helping students understand the nature and value of professional and ethical responsibility, in addition to building on their professional identity as part of preparing them for the engineering profession and practice.

The goal of this paper is not to criticize existing approaches to the teaching of engineering ethics, nor to present an immediate solution to the question of how to better foster ethical development in student engineers. Rather, it is to offer a different approach to add to the collective effort of many to improve engineering ethics education. Our proposed model is theoretical and requires rigorous research and implementation. We predict classes with real life scenarios and open-ended questions promoting discussion will provide students with a more complete exposure to engineering ethics, while at the same time raising the profile of engineering ethics. Second, when introduced to real life scenarios, students can exercise ethical behavior influenced by other motivational forces, such as salience of personal gain, situational cues, and concrete consequences (FeldmanHall et al. 2012). Lastly, considering the spheres of control and person and thing orientation, our new motivational model could contribute to understanding how personality differences influence the decision-making process under both real and hypothetical conditions. It is our hope that this model will stimulate research that will inform pedagogy.
